# Genotyping-by-sequencing and weighted gene co-expression network analysis of genes responsive against *Potato virus Y* in commercial potato cultivars

**DOI:** 10.1371/journal.pone.0303783

**Published:** 2024-05-24

**Authors:** Zahra Hajibarat, Abbas Saidi, Mehrshad Zeinalabedini, Ahmad Mousapour Gorji, Mohammad Reza Ghaffari, Vahid Shariati, Rahim Ahmadvand

**Affiliations:** 1 Faculty of Life Sciences & Biotechnology, Department of Cell & Molecular Biology, Shahid Beheshti University, Tehran, Iran; 2 Department of Systems and Synthetic Biology, Agricultural Biotechnology Research Institute of Iran, Agricultural Research, Education and Extension Organization (AREEO), Karaj, Iran; 3 Department of Vegetable Research, Seed and Plant Improvement Institute (SPII), Agricultural Research, Education and Extension Organization (AREEO), Karaj, Iran; 4 National Institute of Genetic Engineering and Biotechnology, NIGEB Genome Center, Tehran, Iran; Nuclear Science and Technology Research Institute, ISLAMIC REPUBLIC OF IRAN

## Abstract

Potato is considered a key component of the global food system and plays a vital role in strengthening world food security. A major constraint to potato production worldwide is the *Potato Virus Y* (PVY), belonging to the genus *Potyvirus* in the family of *Potyviridae*. Selective breeding of potato with resistance to PVY pathogens remains the best method to limit the impact of viral infections. Understanding the genetic diversity and population structure of potato germplasm is important for breeders to improve new cultivars for the sustainable use of genetic materials in potato breeding to PVY pathogens. While, genetic diversity improvement in modern potato breeding is facing increasingly narrow genetic basis and the decline of the genetic diversity. In this research, we performed genotyping-by-sequencing (GBS)-based diversity analysis on 10 commercial potato cultivars and weighted gene co-expression network analysis (WGCNA) to identify candidate genes related to PVY-resistance. WGCNA is a system biology technique that uses the WGCNA R software package to describe the correlation patterns between genes in multiple samples. In terms of consumption, these cultivars are a high rate among Iranian people. Using population structure analysis, the 10 cultivars were clustered into three groups based on the 118343 single nucleotide polymorphisms (SNPs) generated by GBS. Read depth ranged between 5 and 18. The average data size and Q30 of the reads were 145.98 Mb and 93.63%, respectively. Based on the WGCNA and gene expression analysis, the *StDUF538*, *StGTF3C5*, and *StTMEM161A* genes were associated with PVY resistance in the potato genome. Further, these three hub genes were significantly involved in defense mechanism where the StTMEM161A was involved in the regulation of alkalization apoplast, the StDUF538 was activated in the chloroplast degradation program, and the StGTF3C5 regulated the proteins increase related to defense in the PVY infected cells. In addition, in the genetic improvement programs, these hub genes can be used as genetic markers for screening commercial cultivars for PVY resistance. Our survey demonstrated that the combination of GBS-based genetic diversity germplasm analysis and WGCNA can assist breeders to select cultivars resistant to PVY as well as help design proper crossing schemes in potato breeding.

## Introduction

Potato (*Solanum tuberosum* L.), belonging to *Solanaceae* family, is an important crop for human nutrition. It is also worth noting that potato produces two to four times more food than cereals and can produce more food per unit of water than any other major crop [1]. Potato was introduced to America in 1621 from Europe and has demonstrated an adaptive ability to grow under equatorial (modern Colombia and Venezuela) and cold conditions (South American and the United Kingdom), providing food security in Asia and South America, etc. [2]. The large germplasm base of potato includes several distinct cultivars due to its adaptability to various growing conditions. However, the limited number of parental cultivars used in the present commercial potato varieties may have resulted in a narrow genetic base.

Potato is vulnerable to a wide variety of fungi, bacteria, nematodes, pests, and viral diseases. Among these, PVY is one of the most dangerous pathogens that can significantly reduce the quantity and quality of potato. The genetic architecture of potato PVY resistance has been shaped by several resistance genes [3–5]. Genetic interactions regulate PVY resistance and have an impact on tuber quality and quantity [6]. Furthermore, potato surveys benefit from the availability of the potato genome and sequencing progress with increasing precision genomic information, pan-genomics [7,8], advanced segregant populations [9,10], and genome editing techniques [11,12]. Tilling, ecotilling, and virus-induced gene silencing techniques have been used to link traits to genetic variants in potato [13]. As a complementary technique to explore gene function, forward genetics employs statistical analysis combining trait values and allelic variation in populations to identify QTL, through QTL mapping and/or genome-wide association studies (GWAS) [14]. In potato, these techniques are contributing to the genetic description of agronomic relationships [15,16]. However, the complexity of the interaction between genetic variants and expression regulators containing structural variations makes it difficult to determine the genetic basis of QTLs.

The most challenging aspect of the research is the characterization of causal variants, the validation of gene functions, and the identification of protein networks. Recent research has demonstrated a correlation between the expression level of allele variants, traits, and expression variance, increasing the confidence in candidate gene detection [17]. A combination of accurate phenotyping, advanced genetic materials, and genomic techniques is considered as an alternative approach to improve the ability to detect genes associated with characteristics. It is anticipated that the widespread use of genotyping-by-sequencing (GBS) tool will reorient molecular breeding programmes from MAS (Marker-Assisted Selection) to GS (Genomics Selection), allowing the full potential of genomics-assisted breeding for crop improvement to be realized.

The GBS technique can be used simultaneously for marker discovery and genotyping, allowing many samples to be multiplexed in order to reduce the cost per sample [18]. A modern genomics approach such as GBS can be used to analyze cultivated potato samples in order to gain insight into the genomic diversity of extant germplasm, to identify historic introgressions and hybridizations, and to identify genes that were targeted during domestication that are responsible for agricultural traits [2,19]. Several studies have identified GBS as a highly effective marker discovery method for barely, wheat, rice, soybean, and maize [20–24]. Recently, GBS, has been utilized to simplify complex genomes namely, cotton and potato [25,26]. It is used as a high-throughput and cost-effective molecular tool for routine improvement and screening in most crop species, particularly those with a large genome [27]. Despite the advantages of this method, the application of GBS has some drawbacks for complex, large, and polyploidy genomes to obtain the aligned alleles in a single locus as well as the large amount of data were missed due to low coverage sequencing. Therefore, the use of molecular markers as complement approach with GBS could be useful in heterozygote genotypes like potato. In addition, gene expression correlations can be described using WGCNA analysis, which is a powerful tool. The WGCNA can be used effectively to narrow down the range of candidate genes and has become a favored and popular technique in exploring hub factors, facilitating the revelation of the core gene networks based on the gene expression patterns. To the best of our knowledge, no studies have been performed and no data have been reported yet on commercial potato germplasm based on genotyping by sequencing and WGCNA to explain the gene networks and molecular regulatory mechanisms that govern the PVY resistance.

In this study, commercial cultivar potato were used as material, and 118343 SNP markers were developed based on the GBS strategy were utilized to analyze the genetic diversity, population structure and WGCNA analysis of PVY resistance in the seeding stage of potato. A combination of approaches, namely transcriptomes and GBS were utilized to identify genomic loci and candidate genes involved in PVY resistance among commercial cultivars. The first stage of the study used GBS to identify and genotype SNPs at a genome-wide scale, characterize genetic diversity and population structure, and develop molecular markers using SNPs and SSRs. During the second stage, gene expression profiles were examined in response to PVY resistance as well as the interaction network of biological pathways contributing to PVY resistance of commercial cultivars was characterized.

## Material and methods

### Sample preparation and sequencing

In our study, we obtained 10 commercially grown potato cultivars from the Seed and Plant Improvement Institute (SPII), Karaj, including Agria, Arinda, Bania, Fontaneh, Jelly, Kaizer, Nilva, Santeh, Savalan, and Esprit. These varieties have a high consumption rate among the Iranian population. The seedlings were grown at a temperature of 25°C throughout the experiment, and leaf tissues were sampled. Immediately after sampling, the samples were placed in aluminum foils and stored in liquid nitrogen at -80°C. The DNA was extracted from 1 g of fresh leaves collected from 14–day–old seedlings using the modified Cetyl Trimethyl Ammonium Bromide protocol (CTAB) method. [28]. The DNA purity and integrity were assessed using a 1% agarose gel and a Qubit 3.0 fluorometer. GBS libraries were constructed using Elshire et al. [29] protocol with minor modifications and genotyped using BGI Genomics China.

### GBS data processing and SNP calling

The raw reads were preprocessed by simultaneously demultiplexing using Illumina Experiment Manager ver, 1.16.0. Raw Illumina sequencing reads are available at the National Center for Biotechnology Information (NCBI) Sequence Read Archive (SRA) database under Accession Number SUB14156832. The quality of the individual fastq files was assessed using FASTQC ver, 0.11.5. The SNP calling was conducted using the stacks software version 2. Briefly, reads were filtered using the process_radtags scripts to remove low quality reads with 0.15% of the length of reads. Then, the reads below 90% of base-call accuracy were discarded. Read depth ranged between 5 and 18 and depth of coverage was 5X.

The clean read were mapped to the most recent reference genome assembly of *S*.*tuberosum* using Bowtie2 ver. 2.3.2 [30]. The obtained Sequence Alignment Map files were converted to binary format and sorted with SAMtools 1.6. The loci were generated from the paired end data and SNP calling was performed using the STACKS software. Finally, population’s module of stacks was utilized to generate a variant call format (VCF) file. The reads were filtered to exclude variants with (minor allele frequency) MAF < 0.05, minGQ 15, using VCFtools. The PGDSpider 2.1.1.5 was utilized to convert the VCF file to other file format for downstream applications.

### Population structure analysis

Population structure analysis in commercial potato cultivars was conducted using Discriminant Analysis of Principal Component (DAPC) with an R package, “adegenet”. The DAPC utilizes k-means clustering of principle components to detect groups of individuals. It is run with different numbers of clusters, each of which generates a statistical model and a related likelihood. Therefore, the best model and number of clusters were identified using the lowest Bayesian Information Criterion (BIC). The fineRADstructure package was used to infer population structure based on nearest neighbor haplotype co-ancestry among potato cultivars. Firstly, the haplotypes output file from populations (Stacks pipeline) was converted to a fineRADpainter input file to reduce the maximum number of SNPs at a locus to 10. Secondly, the loci were re-ordered with the script provided. Then, the co-ancestry matrix (RADpainter) was calculated and the individuals were assigned to the cultivars. Finally, the coalescence tree was constructed (finestructure). The outputs were loaded into the program Finestructure GUI for visualization.

### Genetic diversity

Popper was utilized to construct the minimum spanning network (MSN) using Bruvo’s genetic distance between the cultivars and to visualize the relationships among all individuals [31]. A principal component analyses (PCA) was applied using the ape package based on the SNP data. The first two principal components were utilized to assess and visualize the distribution of the individuals using ggplot2 package. According to PCA results, the genetic diversity statics were determined. A Nei genetic distances between individuals were calculated in R using the poppr package [31] and visualized using a heatmap. A neighbour‐joining phylogenetic tree based on a simple matching dissimilarity coefficient. The DAPC, MSN, PCA, and fineRADstructure were utilized as complementary assessments of the population structure.

### SSR identification and primer design

The GBS of 10 potato cultivars was performed with Illumina. The assembled sequences, totaling 1.4 G bases, were utilized in this survey to characterize the distribution of microsatellite in the potato genome. Using VCFtools file, we assembled the whole genome sequence (WGS) by building the consensus map of the variant-file (generated by the freebayes software). Each consensus sequence resulting from the VCFtools was then screened for simple sequence repeats (SSRs) using MISA with default parameters. The acquired SSRs were considered to only represent those containing perfect repeats of SSRs whose basic motifs ranged from 2 to 6 bp with defined minimum repeat units of six iterations for dinucleotide repeats and four iterations for tri-, tetra-, penta-, and hexanucleotide repeats. The SSR search criteria were conducted based on perfect di-, tri-, tetra-, penta-, and hexa-nucleotide motifs minimum number of six, five, four, four, four, and four repeats, respectively. Primer3 v2.23 was used to design primers in the flanking regions of the SSRs. The primers were designed for these markers using Primer3 tool. In total, 3962 primers were identified based on the GBS data and the number and types of identified SSRs were detected. Putative SSR markers were selected based on the following parameters: primer length between 22–25 bp, PCR product length between 100 and 250 bp, primer melting temperature (Tm) between 45–65 °C with an optimum of 55 °C, and GC content of 40–70%.

### WGCNA analysis

For this survey, raw microarray data related to transcriptome profiling in *S*.*tuberosum* for PVY infection was retrieved from the GEO microarray database with Agilent-015425 PSUMAC-Solanum tuberosum-44K (Probe Name version). The dataset consisted of four time points of 0, 1, 3, and 6 days after PVY infections with four replicates for GSE46180 including GSM1125694, GSM1125695, GSM1125696, GSM1125697, GSM1125698, GSM1125699, GSM1125700, GSM1125701, GSM1125702, GSM1125703, GSM1125704, GSM1125705, GSM1125706, GSM1125707, GSM1125708, GSM1125709, GSM1125710, GSM1125711, GSM1125712, GSM1125713, GSM1125714, GSM1125715, GSM1125716, GSM1125717, GSM1125718, GSM1125719, GSM1125720, GSM1125721, GSM1125722, GSM1125723, GSM1125725, GSM1125726, GSM1125728, GSM1125730, GSM1125731, GSM1125733, GSM1125734, GSM1125736, GSM1125737, GSM1125738, GSM1125739, GSM1125740, GSM1125741, GSM1125742, GSM1125743, GSM1125744, GSM1125745, GSM1125746, and GSM1125747.

To convert probe set to gene IDs, DAVID site (https://david.ncifcrf.gov) was used. The genes were filtered to eliminate low-expressed or outlier genes. Then, the remaining genes were utilized to gene co-expression network analysis. The differentially expressed genes (DEGs) were identified with the criteria of p<o.o5 and log2 FC ≥ 2. Genes with log2 FC> 2 and log2 FC< -2 were detected as up- and down-regulated DEGs, respectively.

Subsequently, WGCNA (Weighted gene co-expression network analysis) was performed to establish the potato DEGs co-expression network using R package (flashClust). The WGCNA was used to detect modules with highly expressed genes, and categorized them with module eigengene (ME). We performed the WGCNA analysis at 3 weeks old potato seedlings stage. The R package WGCNA (v1.61; https://cran.r-project.org/web/packages/WGCNA/index.html) was employed for the analysis of DEGs’ co-expression module. A total of six WGCNA modules (co-expression network) of eigengenes were identified. Correlation of networks with PVY resistance was drawn with the criterion of stability correlation of p≤0.05. The modules with gene significance (Pearson’s correlation coefficient) ≥0.6 for PVY infections (infection at the 0, 3, and 6 days in leaf tissues) were retained for further analyses.

### GO Ontology

Using Blast2GO, the GO analysis was performed using classification of DEGs indicating probable pathways captured by genes involved in biological processes, molecular functions, and cellular components with the criterion of p< 0.05.

### Validating hub genes

Hub genes within the modules were detected using module membership (MM) which was calculated based on Pearson correlations between the expression level and module eigengenes. A relatively high MM indicated that hub genes have a relatively high correlation between individual gene and the module eigenegene. In each module, the genes with eigengene-based connectivity value (|KME|) > 0.9 and topological overlap measure (TOM) value > 0.2 were regarded as hub genes. Based on the hub gene correlation network and their high connectivity, three genes including domain of unknown function 538 (StDUF538), transmembrane protein 161A (StTMEM161A), and general transcription factor 3C polypeptide 5-like (StGTF3C5) were selected.

### Plant growth and PVY inoculation

To investigate the reaction of 10 cultivars to Potato Virus Y (PVY), leaves were mechanically inoculated under greenhouse conditions with three replications according to the standard protocol of international potato center (CIP) (CIP, 2007). Three and six days post-inoculation, virus infection was monitored with local symptoms. The infected leaf and tissues were collected at the 0, 3, and 6 days post inoculation from potato seedlings in the greenhouse at -70^0C^

### RNA isolation and qRT-PCR

RNX-Plus protocol was utilized to isolate mRNA. The concentration and quality of RNA was determined using the Nanodrop. Synthesis of cDNA was performed using one-step reverse transcription kit (Easy cDNA Synthesis Kit, Iran). Suitable primer was designed and the potato StEF-1α gene was utilized as an internal control for the performance of qRT-PCR. All primers utilized in the gene expression analysis are listed in S1 Table. Analysis of gene expression was performed using the 2^-ΔΔCt^ method.

### Characterization of morphological and physiological indices

Four morphological traits were measured in this survey including plant height, stem number, fresh weight, and dry weight under 0, 3, and 6 days after PVY infection. To investigate physiological traits, healthy and infected potato leaves were collected at the seedling stage. Fresh leaf samples were washed by distilled water in the laboratory. Then, they were kept to dry at room temperature (18°C) and analyzed for the determination of chlorophylls (Ch-a and Ch-b) and carotenoids content. Chlorophylls (Ch-a and Ch-b) and carotenoids content were measured using the protocol by Sumanta et al. [32]. The solution mixture was analyzed for Ch-a and Ch-b and carotenoids content in spectrophotometer with 663, 645, and 470 wavelengths. The following equation was used for the quantification of Ch-a, Ch-b, total chlorophyll, and carotenoids by

$$\text{Ch-a}=12.25{\text{A}}_{663.2}–279{\text{A}}_{646.8}$$

$$\text{Ch-b}=21.5{\text{A}}_{646.8}–5.1{\text{A}}_{663.2}$$

$${\text{C}}_{\text{x}+\text{c}}=\left(1000{\text{A}}_{470}–\;1.82{\text{C}}_{\text{a}}–85.02{\text{C}}_{\text{b}}\right)/198$$

$$\text{Total}\;\text{chlorophyll}\left(\text{mg/g}\right)=\left[20.2\;(\text{A}645\right)+8.02(\text{A}663)]$$

A=Absorbance,Ch-a=Chlorophylla,Ch-b=Chlorophyllb,Cx+c=Carotenoids

### Statistical analysis

Using the SPSS software, statistical analysis was performed and differences across tissues were measured using the one-way ANOVA test. *Significant* differences among group means were calculated at 5% level.

## Results

### SNP calling, data filtering, and Marker qualify

**Fig 1:**
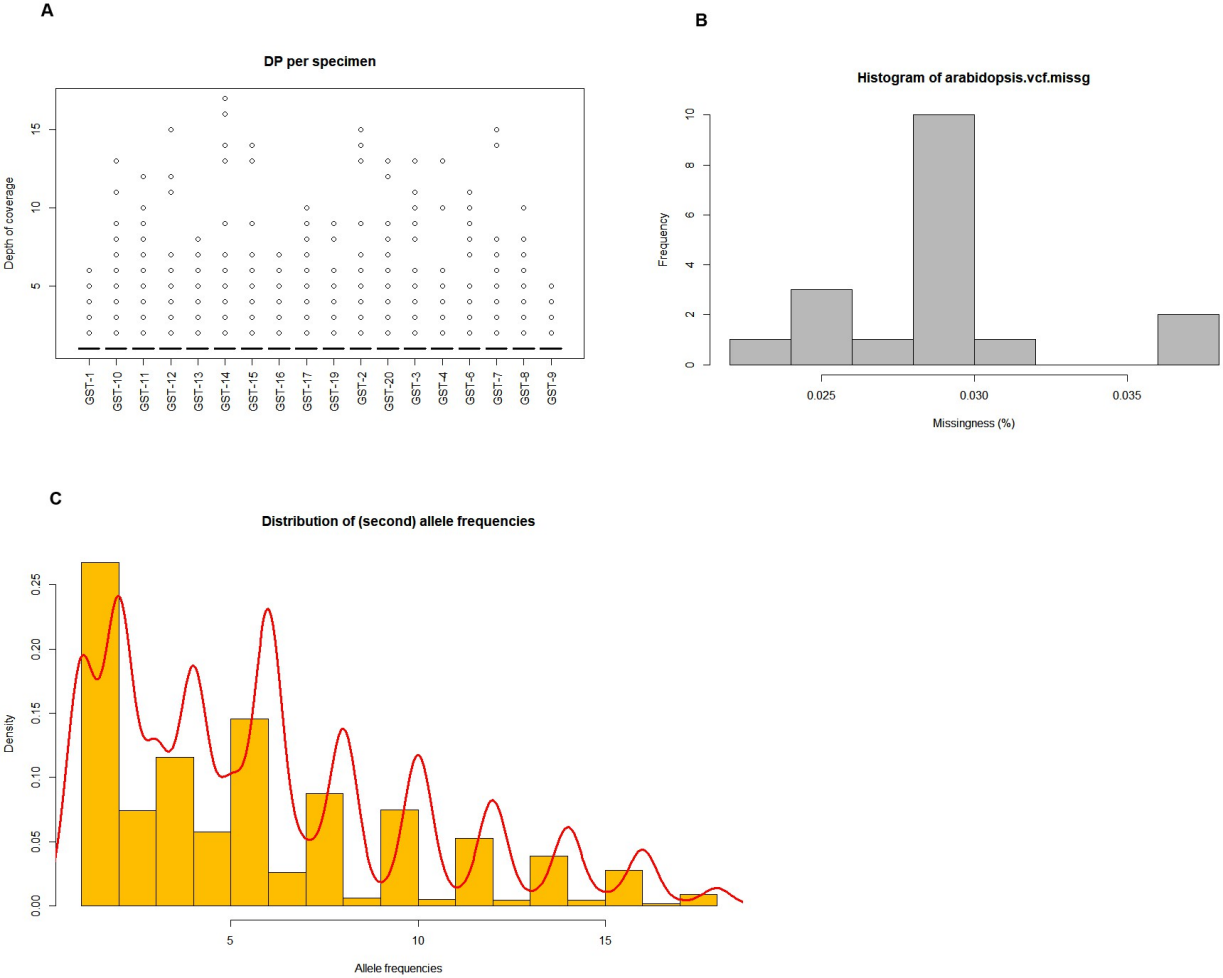
Distribution of (a) Read depth, (b) Missing data, and (c) Minor allele frequency (MAF) for SNP markers in potato cultivars.

The SNP genotyping of the cultivars generated a total of 118343 (SNP) markers. The markers included 0 to 3.5 percentage missing data. Read depth ranged between 5 and 18. The distribution of read depths revealed that most markers had read depth between 5 and 8 (Fig 1A and 1B) and the minor allele frequency (MAF) varied from 0 to 0.27. After filtering for read depth, quality, and missing information, clean reads were obtained from the potato cultivars with an average of thousands real per sample. The average data size and Q30 of the reads were 145.98 Mb and 93.63%, respectively. Of the 2.63 million SNPs identified in these loci, 118343 remained and were used in the subsequent analyses (Fig 1C).

### Population structure and germplasm clustering

**Fig 2:**
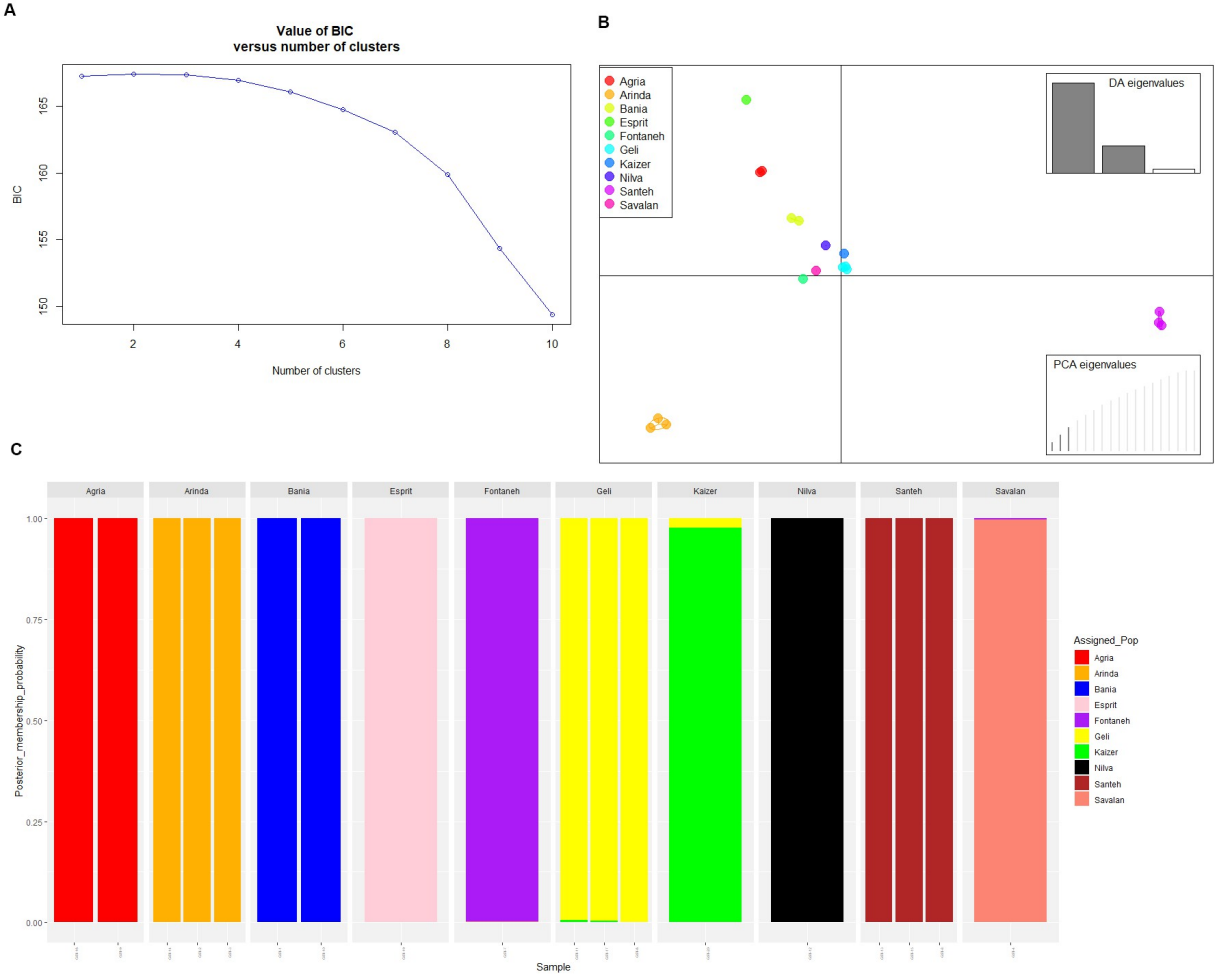
Discriminant analysis of principal components (DAPC) for 10 potato cultivars using SNPs. (**A**) Statistical determination of the optimum number of clusters, (**B**) Scatter plot from the DAPC analysis, (**C**) and Complete picture of membership probability information of all individuals.

Variation among the genetic clusters was represented in DAPC using three principal components (PCs) and two discriminant functions (DA eigenvalues). Tree distinct genetic clusters of 10 cultivars were positioned in distinguished quadrants (Fig 2). Also, three clusters were detected in coincidence with the lowest Bayesian Information Criterion (BIC) value using *find*.*clusters* function (Fig 2A). Further, the results were visualized as two-dimension scatter plot. Membership coefficients of the cultivars to each group were between 0.998 and 1, therefore confirming that there was a very low admixture with a structured population. Exceptions to these values were cultivar Kaizer and Jelly whose values were 0.98 and 0.988, respectively.

**Fig 3:**
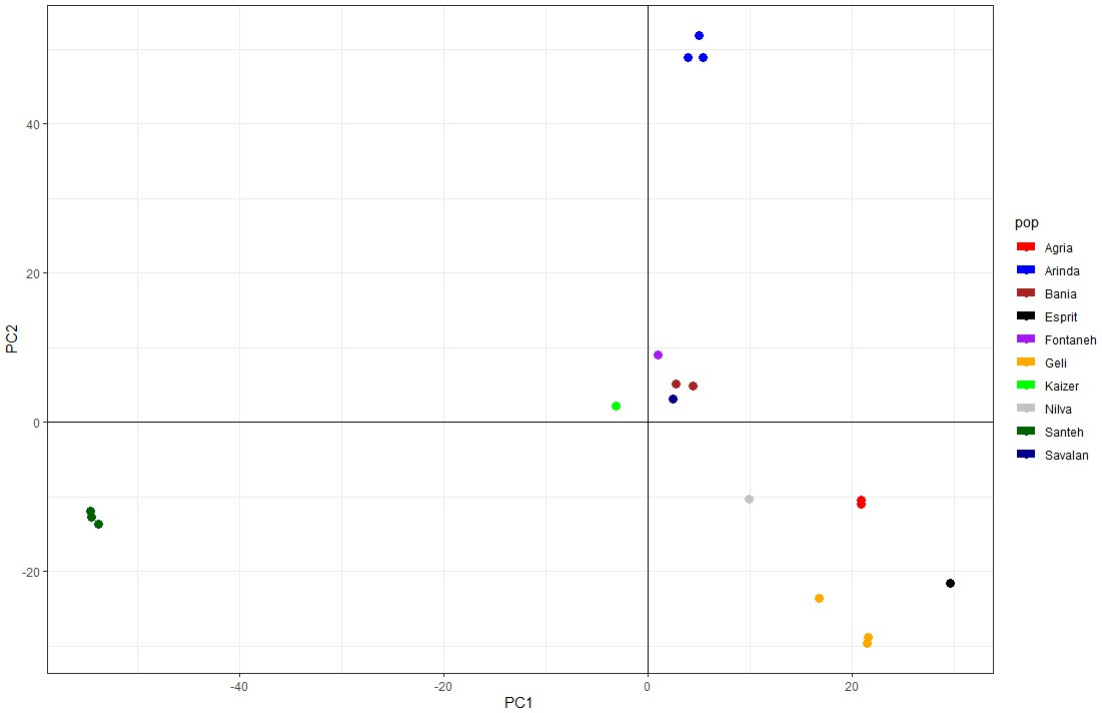
Principal component analysis (PCA) scatter plot based on 114824 GBS-obtained SNPs on evaluated cultivars of *S*.*tuberosum*.

Three first PCs (29% of variance conserved) of PCA and three discriminant eigenvalues were retained. In Fig 2B, DAPC was grouped into three clusters such as cluster I (Agria, Bamba, Fontaneh, Jelly, Kaizer, Milva, and Savalan), cluster II (Sante), and cluster III (Arinda). The DAPC analysis of subpopulations showed that Esprit is more distant from the others in three clusters (Fig 2B and 2C). The PCA plot according to the commercial matrix 114383 SNPs reflected the most likely phylogenetic relationships. Individuals of *S*.*tuberosum* from the commercial cultivars were clustered in three main groups (Fig 3). Based on the PCA analysis, three clusters composited cluster I (Arinda, Fontaneh, and Bamba), cluster II (Agria, Milva, Jelly, and Esprit), and cluster III (Kaizer and Sante).

### Genetic distance

**Fig 4:**
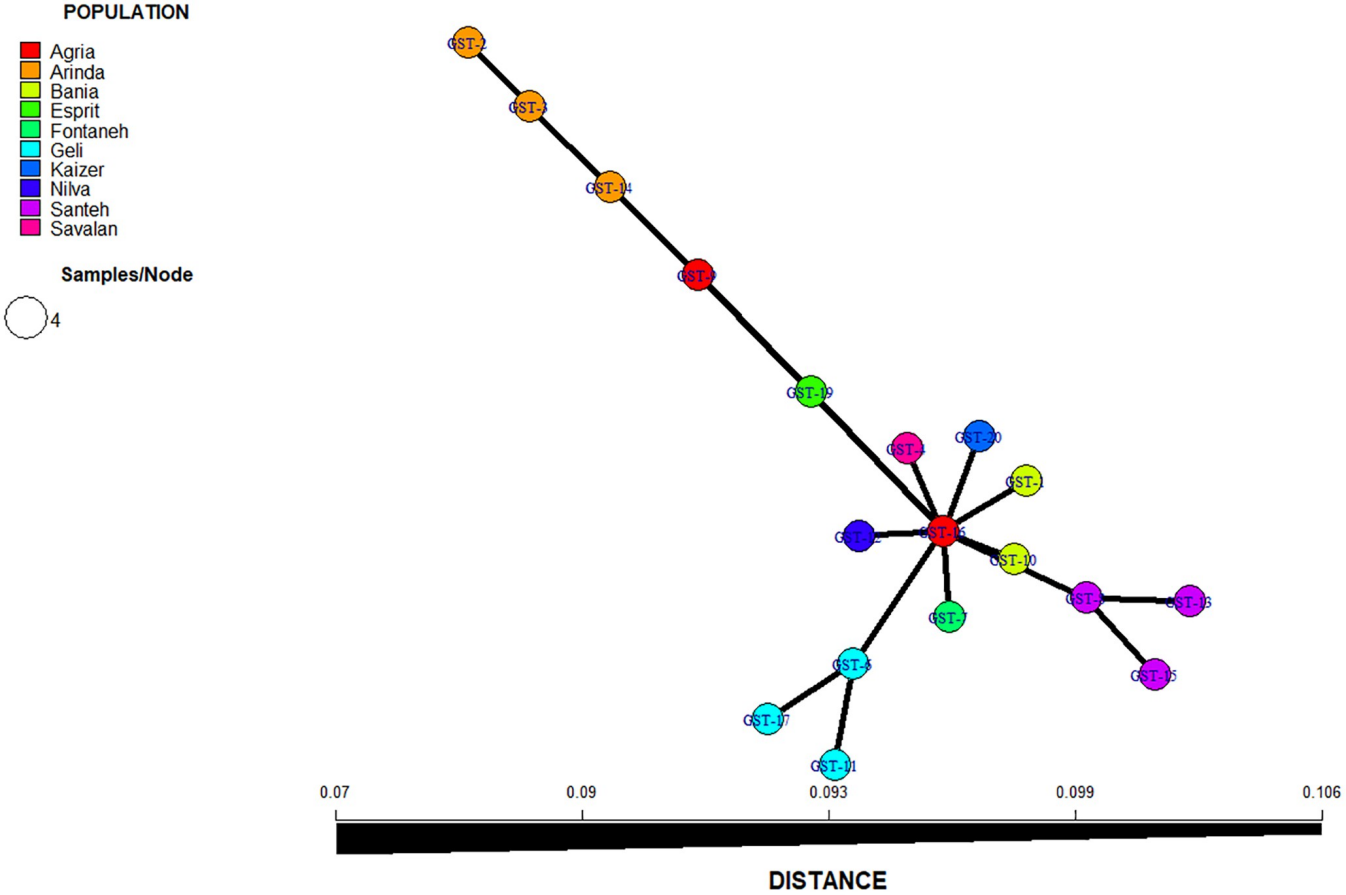
Minimum spanning network showing the differences among the genotypes. Each circle represents a unique genotype (shown as pies).

In the MSN, tree was constructed from the SNP dataset and individuals were clustered neatly by cultivars. Based on this method, the cultivars were categorized into three clusters according to Bruvo’s distance. These clusters included, cluster I (Agria, Arinda, Bamba, Savalan, Milva, Kizer, Fontaneh, and Esprit), cluster II (Sante), and cluster III (Jelly). The Agria may represent the ancestral cultivar. Further, the MSN did not form a ring-shaped network, showing that hereditary population structure was weak. The reticulated network (Fig 4) showed patterns corresponding with MSN resulting from a discriminant analysis of principal components.

**Fig 5:**
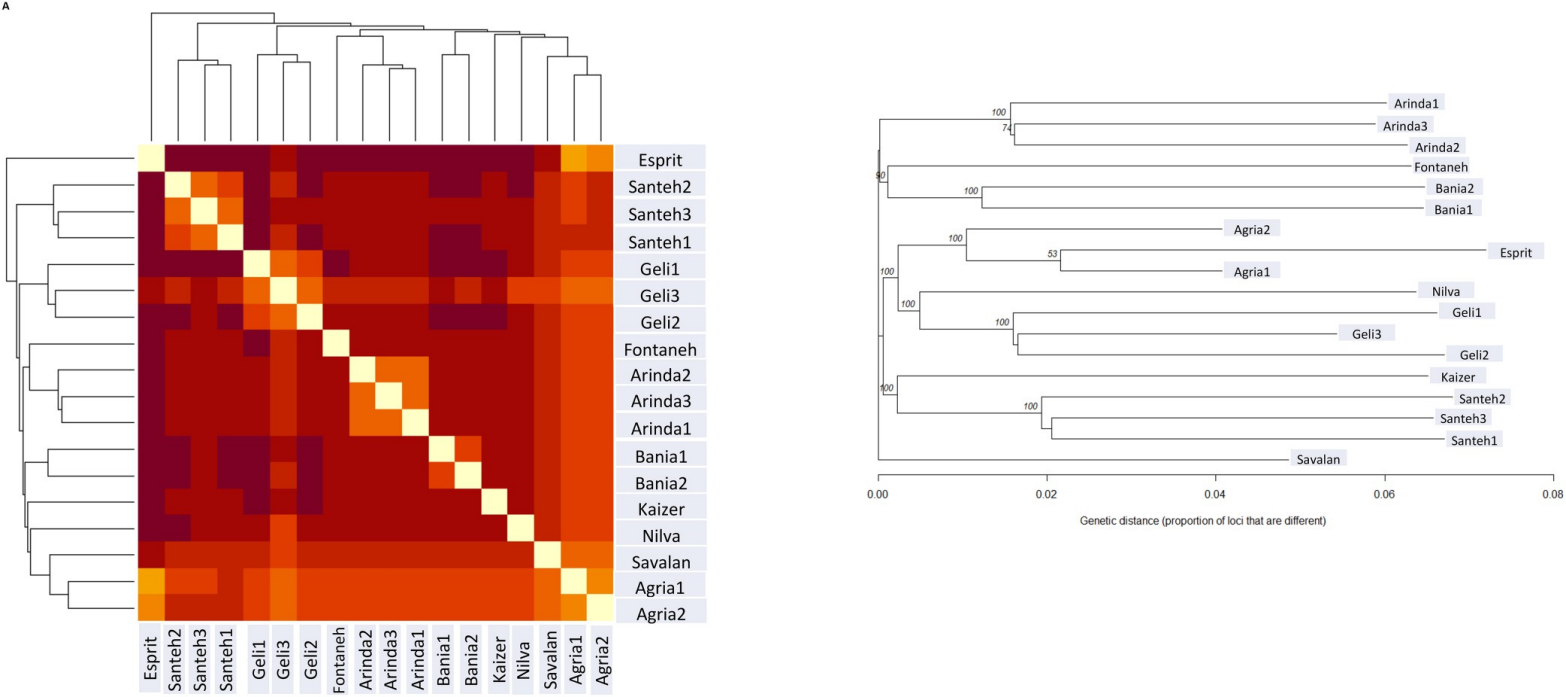
A) Heatmap of genetic distances between 10 potato cultivars. Heatmap illustrates genetic relationships from lower (yellow/white) to higher (red/orange) among individuals of 10 accessions. B) Neighbour joining tree based on Edward’s distance calculated for 10 accessions of potato.

To confirm the distribution of individuals into the clusters by DAPC analysis, a Nei genetic distance matrix was calculated. To obtain a more complete picture of the relationships among the 10 potato cultivars, a neighbour joining (NJ) tree was constructed based on Edward’s distance. The dendrogram was constructed using Nei genetic distance among individuals of the whole population. The individuals were grouped into three clusters containing cluster I (Arinda, Fontaneh, and Bamba), cluster II (Agria, Milva, Esprit, and Jelly), and cluster III (Kaizer and Sante) in the population. The Nei based GDs between populations were calculated and visualized. Based on the GD values, the different cultivars were classified into three groups including cluster I (Agria, Savalan, Milva, Kaizer, Bamba, Arinda, and Fontaneh), cluster II (Jelly), and cluster III (Sante). Further, Esprit is as an out-group among 10 cultivars (Fig 5A). The results from both the PCA and DAPC analyses showed that there was a low admixture between the cultivars.

### Genetic structure of potato cultivars

**Fig 6:**
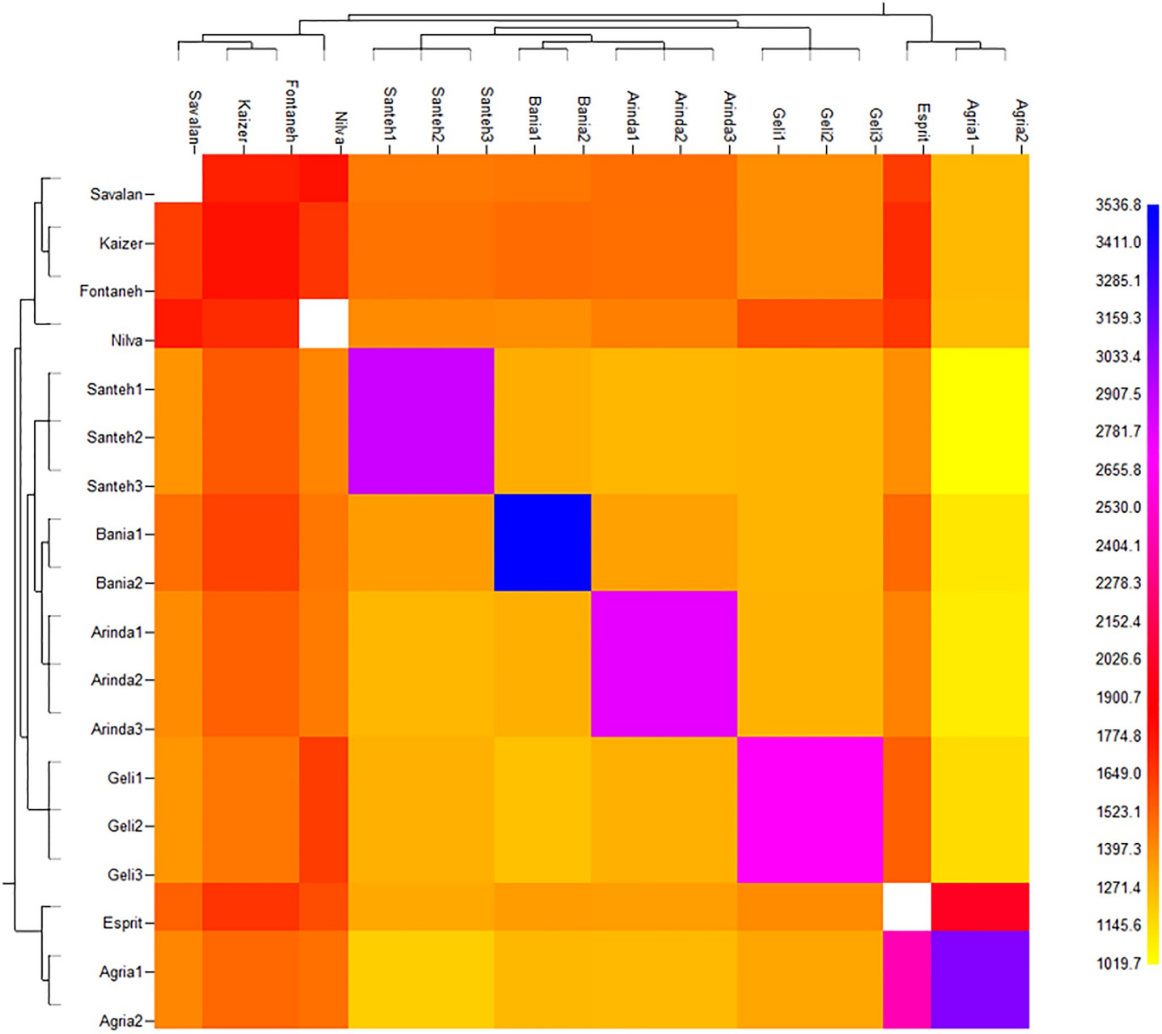
Clustered fineRADstructure co-ancestry matrix of *S*.*tuberosum* estimated from the commercial potato cultivars. Individuals from the three groups cluster. Hybrid individuals are clearly identifiable, sharing relatively more equal coancestry levels with both group I and III.

Our results revealed that the fineRADstructure matrix estimated lower co-ancestry (and hence higher genotypic variation) for the group I as compared to group III. The presence of hybrid of group of I and III revealed evident signs of population admixture in two groups. The FineRADstructure analysis based on the nearest neighbor haplotype or coancestry confirmed the admixture pattern. All cultivars were related to three main ancestral populations (Fig 6). Based on to the fineRADstructure results (Fig 6), the three genetic clusters shared more co-ancestry within each other than between them. Colors indicate scale of relatedness between individuals, with yellow being low relatedness and blue/black indicating high relatedness. Inferred tree relating cultivars is shown in Fig 6 with posterior assignment probabilities. For each row, cell color indicates lowest probability of coancestry, and red and blue indicate higher probabilities. The Fig 6 showed a clear split between three groups, as well as an indication of within species substructure. The FineRADstructure detects and reports all extra sample relationships that ascents above the statistical noise in the co-ancestry matrix. Comparison between groups showed that individuals of *S*.*tuberosum* from group III share more co-ancestry with individuals from group II, than with group I (Fig 6). Further, genetic hybrids between the three groups are clearly detected by the fineRADstructure. The co-ancestry matrix showed that Esprit is a hybrid. It has received a remarkable number of chunks from the Agria, Jelly, Arinda, Bamba, and Sante cultivars. Also, the co-ancestry indicated that Milva is a hybrid between the cultivars of groups III (Fontaneh, Kaizer, and Savalan) and II (Jelly, Arinda, Bamba, and Sante). The boxes in Fig 6 indicate evident signs of admixture. It seems that the two subgroups (Bamba and Arinda) were evidently admixed with Sante and Jelly. Interestingly, signs of admixture can also be seen between Milva and Savalan (group II) subgroups of the chunks were estimated to be imported from Esprit cultivar. It is significantly higher than imported into the other subgroups in group II. Signs of admixture in the three groups are indicated. Among three groups, group III had strong admixture as compared to groups I and II. Esprit revealed stronger signatures of population admixture. Compared with subgroup (group I), the signature of population admixture was weaker in the purely I subgroup, Agria. These findings revealed that cultivars were divided into groups of individuals with indistinguishable genetic ancestry in the dataset.

### Simple sequence repeat (SSR) types in the potato genome

A total of 3962 primer pairs were successfully developed. Among the SSRs, dinucleotide microsatellites were the most abundant (73.22%), followed by tri- (15.65%), tetra- (8.57%), penta- (2.26%), and hexanucleotide (0.55%) types. Among the total dinucleotide motifs, 13531 AT (37.323%) and 13015 TA (35.90%) dinucleotide motifs were the most detected types, while only ATTTTT and AAAATA motifs were the lowest SSRs available in the GBS data. Among the tri-nucleotide, only AAT (1.779%) by GAA (1.87%), ATT (1.779%), TTC (1.74%), AAG (1.69%), TCT (1.48%), CTT (1.35%), TTA (1.29%), ATA (1.15%), and TGT (1.14%) motif types were identified (Table 1). As shown in Table 2, some of the newly developed SSR loci based on GBS were randomly selected from the set of the *in silico* detected polymorphic SSR loci. Based on our results, two novel sets of molecular markers namely SSR and SNP markers were derived as GBS data. These novel genetic markers could be used to generate a dense genetic map of potato.

**Table 1:** Summary of potato SSRs detected based on GBS sequences.

Loci	Primer sequence (5′–3′)	Repeat motif	Product size	Tm
ABRII-570	F:TCATGGGGTGTTCGAATTTGT	(TA)7	250	58.13
	R:GTCAAGCTTTGTGTGGTTGGTAA			
ABRII-3459	F:ACTCCACCAAGTTTGCCTGAT	(AT)7	250	59.854
	R:ACGTTGCAAGTTCCAGTTCTT			
ABRII-24822	F:TGCCCACACCATCGAAAAGA	(AT)10	230	59.89
	R:TCTCTCATGTCCCGAGGATGA			
ABRII-21242	F:TCTCCAAGCCCTAGCGTACT	(GACTTG)5	220	60.033
	R:CGCGATTCCGAGTTCGTATG			
ABRII-27029	F:AACCTCAAGCGGCTCTTCTC	(CT)6	210	60.037
	R:GGAGTTTGGCGATCCGAAGA			
ABRII-20814	F:GCCACTCACGGACCCATAAA	(TCTT)4	200	60.036
	R:TGAGGCTCGACACTGGTAGA			
ABRII-20943	F:ACACGCACCCATCTGCATAA	(TATTA)4	200	60.036
	R:TGTGAGTGGAGTGGGGTAGT			
ABRII-331	F:GCCGACAGAGTTCAAGGGTAA	(TC)6	197	59.998
	R:AACATTGTTGGGTGCGAAGA			
ABRII-14492	F:GGCCAAGCGTTTGTGTTCTT	(TTA)6	196	59.898
	R:GTGGTGGTGTGTTCGGAGAA			
ABRII-14810	F:GCGAGCTGCAGTCATTTGTT	(AATT)4	196	59.761
	R:GTCGCCATTCGCCATTTTGT			
ABRII-27697	F:GAGCACCGAGTTGGGTTAGA	(AT)7	194	59.393
	R:CGTTGCAAGGGTCCATCCTA			
ABRII-2517	F:TTCCGCGAAATCAGCCCTTA	(TA)12	193	59.749
	R:CTTCCTCACGCGCCTAGTAG			
ABRII-3987	F:CGTTGGCCCTTCACTCGATA	(GA)7	186	59.825
	R:TCCAACACCTGCACCAACAA			
ABRII-25850	F:GGTGTGAAATGGGCGGGTTA	(AT)8	185	60.61
	R:GCTGCCCTCTTGGTCATCAT			
ABRII-3504	F:GATCGCCGGATCGTTCATTT	(TA)7	184	58.782
	R:CAACCATGCCCCCAACCTAA			
ABRII-28420	F:GCGGCTCAAGCACACTAGTA	(AT)8	180	60.109
	R:GGTGGGTGCTTAAGGCTCTT			
ABRII-815	F:AGACAAGTGAGGCCCCATTG	(TA)6	179	59.961
	R:CATGCAAACCGCGAACCATA			
ABRII-28254	F:ACAACTTGACGTGATGCATTCA	(TA)9	175	59.122
	R:GTGGAGACATTAGGGCCTGTA			
ABRII-3598	F:AGGTCCATTCGGCGCAATAA	(TAA)7	174	60.107
	R:GCGAGTCACCAAACGGTTTC			
ABRII-28678	F:CGAAGCATCCGGGGTAAAGT	(TA)9	170	60.108
	R:CTCGGCTCAAGTGCAACAAA			
ABRII-3016	F:CTCTCCCGCCATGCCATTAT	(AT)14	169	59.963
	R:GTCTGGCTGGCCCATCTAAC			
ABRII-28680	F:GGGCCAAAAGCTGCTGATTT	(TAT)5	165	59.677
	R:AGGCGGGATTACAGCGAAAA			
ABRII-6390	F:GGTGTTCACGGGTTGGTTTG	(AAACC)4	164	59.898
	R:CAAGCCAAACCGAAACCGATA			
ABRII-17255	F:ATGTCCTCCGTGCACAACTT	(CAT)5	160	59.891
	R:TTGGGCCCTAGCTAGCTACA			
ABRII-17677	F:GCTGAGCAACTTCGCACATT	(ATA)7	160	59.761
	R:TTGCCCCCATTGCAATTCTC			
ABRII-12206	F:TGCCTCACTTTGTGCCTCTT	(AAAGA)4	156	59.817
	R:CACACACCCTTGCCCAATTG			
ABRII-13611	F:CTGCCCATTCCCCGTTTACT	(TATT)4	156	60.034
	R:TGCCTAGGGGAAGCCATACA			
ABRII-18747	F:CGTCAGATGCACCGGGATAG	(GTTA)5	156	60.319
	R:ACTCTTGACAGCGCCATTGA			
ABRII-449	F:GGATGAGCCATGCAGCTACT	(TAT)9	149	59.892
	R:GGGCATCCAATCACCCTCAT			
ABRII-549	F:TGAGGCCTTCGGTTGAGTTT	(TAA)5	149	59.528
	R:GGAGACCACACCAACCATTCT			

**Table 2:** Frequency distribution of different SSRs based on motif type.

SSR motif	Number of SSRs	Motif frequency (%)
AT	13531	37.3238077
TA	13015	35.9004772
AAT	782	2.15706286
GAA	680	1.87570684
ATT	645	1.7791631
TTC	631	1.74054561
AAG	614	1.69365294
TCT	537	1.48125672
CTT	490	1.35161228
TTA	468	1.29092765
ATA	417	1.15024963
TGT	414	1.14197446
AAAT	826	2.27843213
TTTA	428	1.18059195
ATTT	416	1.14749124
TATT	283	0.78062505
AATA	266	0.73373238
TTAT	234	0.64546382
TAAA	212	0.58477919
ATAA	173	0.47720189
TTAA	166	0.45789314
AAACC	144	0.39720851
AAAAT	118	0.3254903
GGTTT	112	0.30893995
TTGAG	81	0.22342979
TTTTA	78	0.21515461
ATTTT	65	0.17929551
TATTT	61	0.16826194
CTCAA	61	0.16826194
TTTAT	52	0.14343641
ACTCA	49	0.13516123
AAAAAT	29	0.07999338
ATTGTG	25	0.06895981
AAGTTC	24	0.06620142
CACAAT	23	0.06344303
TTGTGA	21	0.05792624
TATTTT	18	0.04965106
TAAAAA	17	0.04689267
GAATAG	17	0.04689267
ATTTTT	15	0.04137589
AAAATA	15	0.04137589

### WGCNA analysis for PVY resistance

**Fig 7:**
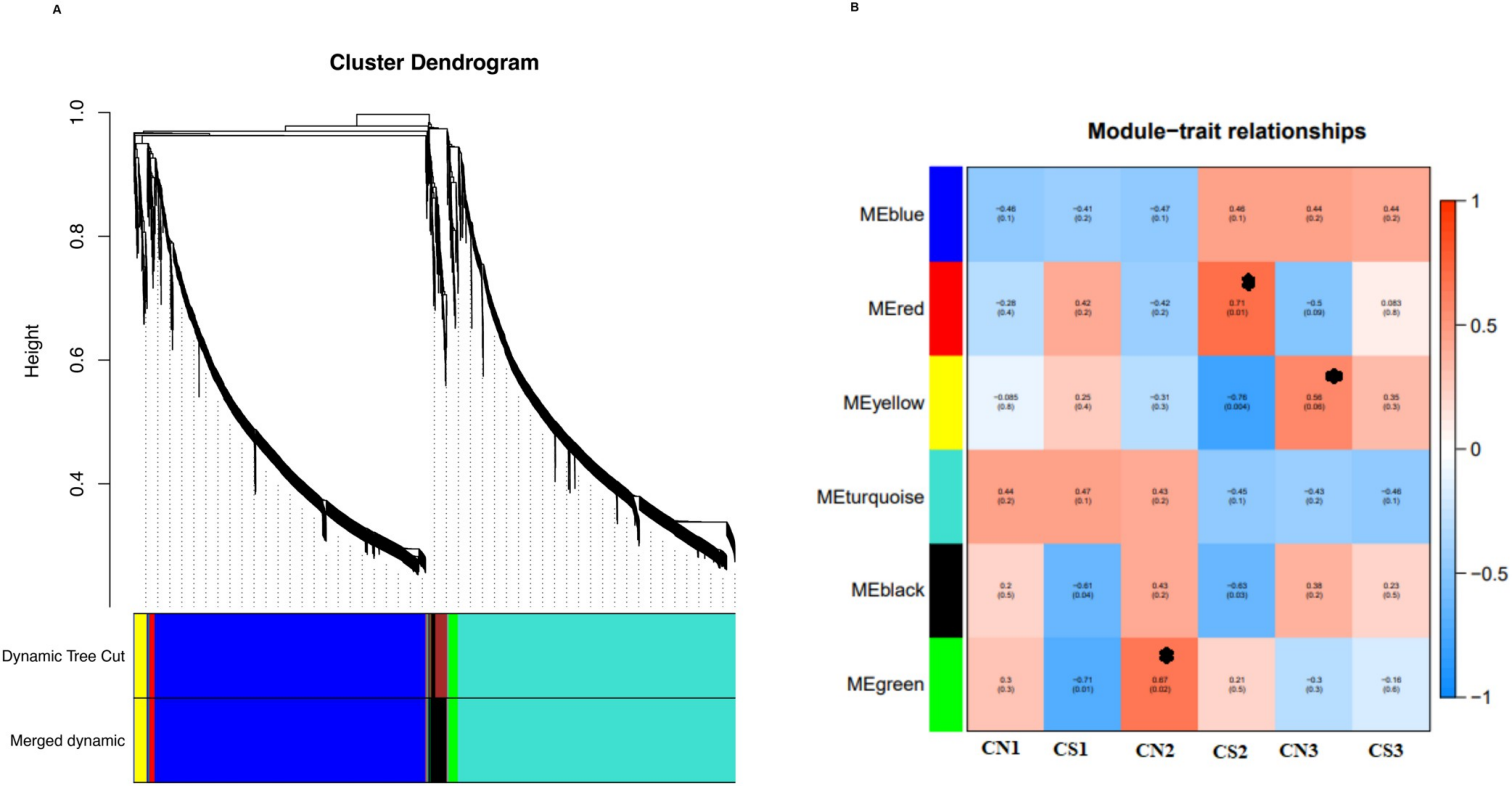
WGCNA module identification and correlation analysis. (**A**) the clustering dendrogram and expression heatmap of genes identifying the WGCNA modules. (**B**) the correlation of the identified modules with the PVY resistance in different treatments. Modules significantly associated with the traits with p value < 0.05, and are indicated by asterisk*. Red and blue color notes positive and negative correlation with gene expression, respectively.

To capture the broader view of the molecular mechanisms driving PVY-resistance, the WGCNA was performed and then combined with a GBS approach. A soft threshold power showed a network with a scale-free topology with R2 > 0.85 for each genotype. In the consensus network, genes were grouped in seven co-expression modules with a size range of 57 (red module) to 106 (yellow module) genes. The PVY-resistance was correlated with three modules (P< 0.05) (Fig 7).

In this survey, three selected genes with a high degree of connectivity, based on the MCC in the yellow, red, and green modules, were correlated with PVY resistance. Based on our results, the *StDUF538* gene had differed expression in the 10 cultivars. The four cultivars (Esprit, Jelly, Kaizer, and Milva) had high transcripts of StDUF538 among cultivars. These findings are associated to three phenotypes (WR, WF, and PH) at the 3 and 6 days PVY infection. To validate the accuracy of the WGCNA data, qRT-PCR was conducted for the three genes: StDUF538, StGTF3C5, and StTMEM161A. According to WGCNA analysis, the first hub gene, *StTMEM161A* gene, possesses a high correlation in the module. In this study, the Bamba and Esprit cultivars had a high relative expression rate as compared to other cultivars under PVY infection. The second hub gene, StGTF3C5, encodes a subunit of the DNA-binding subcomplex (TFIIIC2) of transcription factor IIIC (TFIIIC) that was identified as a hub gene in the module. The third hub gene, StDUF538 gene, is another hub gene that is differentially expressed gene among the 10 cultivars under PVY infection. The relationship of StDUF538, gene expression level with the chlorophyll content of the PVY-infected leaves is probably resulted in response to biotic stress. Further, the *StTMEM161A* and *StGTF3C5* genes are fundamental mediator in the pH apoplast and regulation of tRNA biogenesis in growth and response to pathogens in the resistant cultivar, respectively.

#### Candidate gene discovery

**Fig 8:**
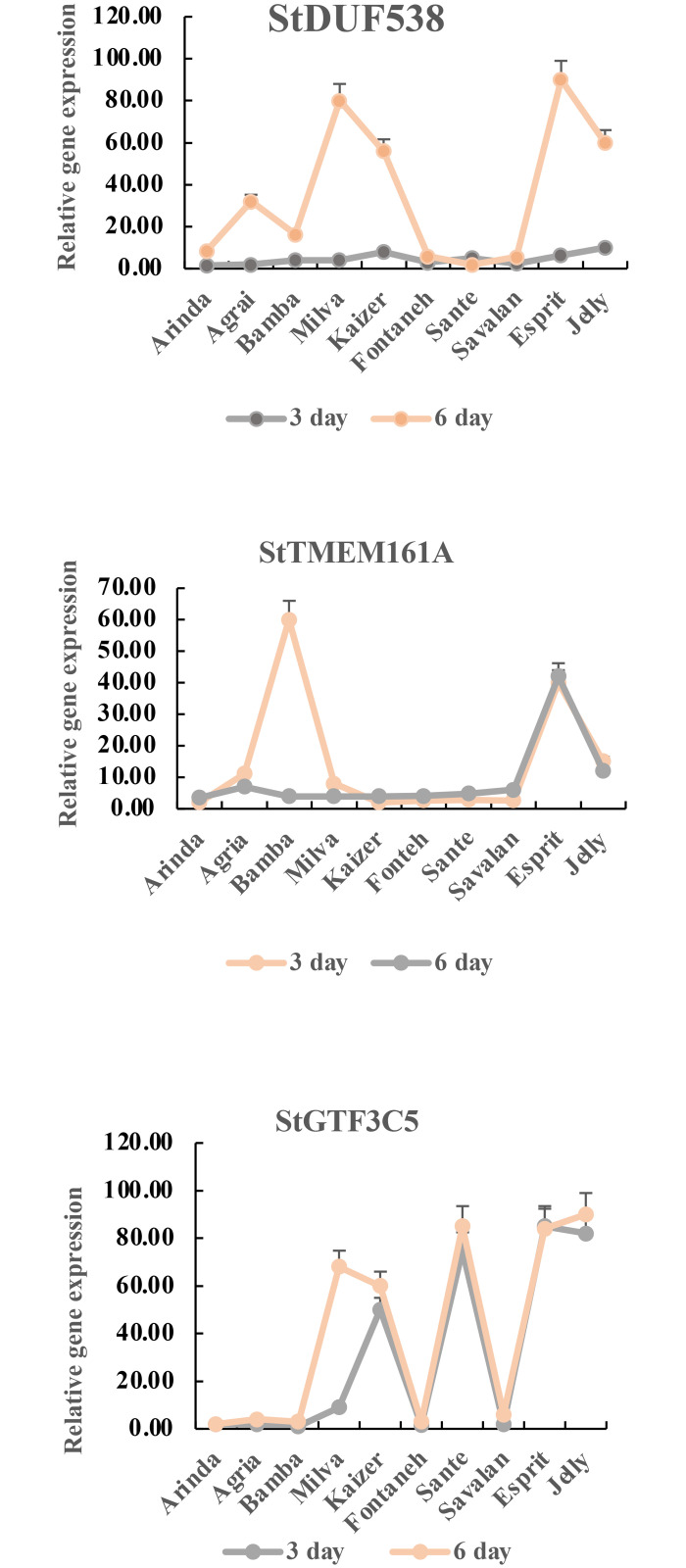
Three hub genes significantly expressed in leaves samples at the 3 and 6 days after the PVY infection.

To further confirm the results of computational analysis, we selected three candidate genes based on the GO analysis results and WGCNA modules. Three genes were related to the PVY resistance in potato. The findings revealed that the expression levels of the three genes (StDUF538, StTMEM161A, and StGTF3C5) in qRT-PCR were consistent with WGCNA. The three candidate genes responsive to PVY were significantly expressed among the 10 cultivars which espciallyin Jelly and Esprit cultivars. Our findings showed that the StDUF538 was increased in four (Esprit, Jelly, Kaizer, and Milva) cultivars. However, it was unaltered in the six (Fonteneh, Savalan, Bamba, Agria, Arinda, and Sante) cultivar leaves. Further, the StGTF3C5 was highly expressed in Esprit, Bamba, and Jelly leaves. However, it did not show high expression in other cultivars. The StTMEM161A was induced differentially by the potato PVY in leaves. The StTMEM161A expression was maximum in five (Esprit, Jelly, Sante, Kaizer, and Milva) cultivars. Its expression in five (Arinda, Agria, Bamba, Fonteneh, and Savalan) cultivars were unaltered. Based on our results, Esprit cultivar had the maximum expression of *StDUF538*, *StTMEM161A*, and *StGTF3C5* genes. Further, Esprit possesses the low chlorophyll content at the 3 and 6 days after PVY infection as well as Esprit had the maximum fresh weight and dry weight at 3 and 6 days after infection. Thus, Esprit can be suggested as a resistant cultivar against PVY infection (Fig 8).

#### Function annotation and classification

**Fig 9:**
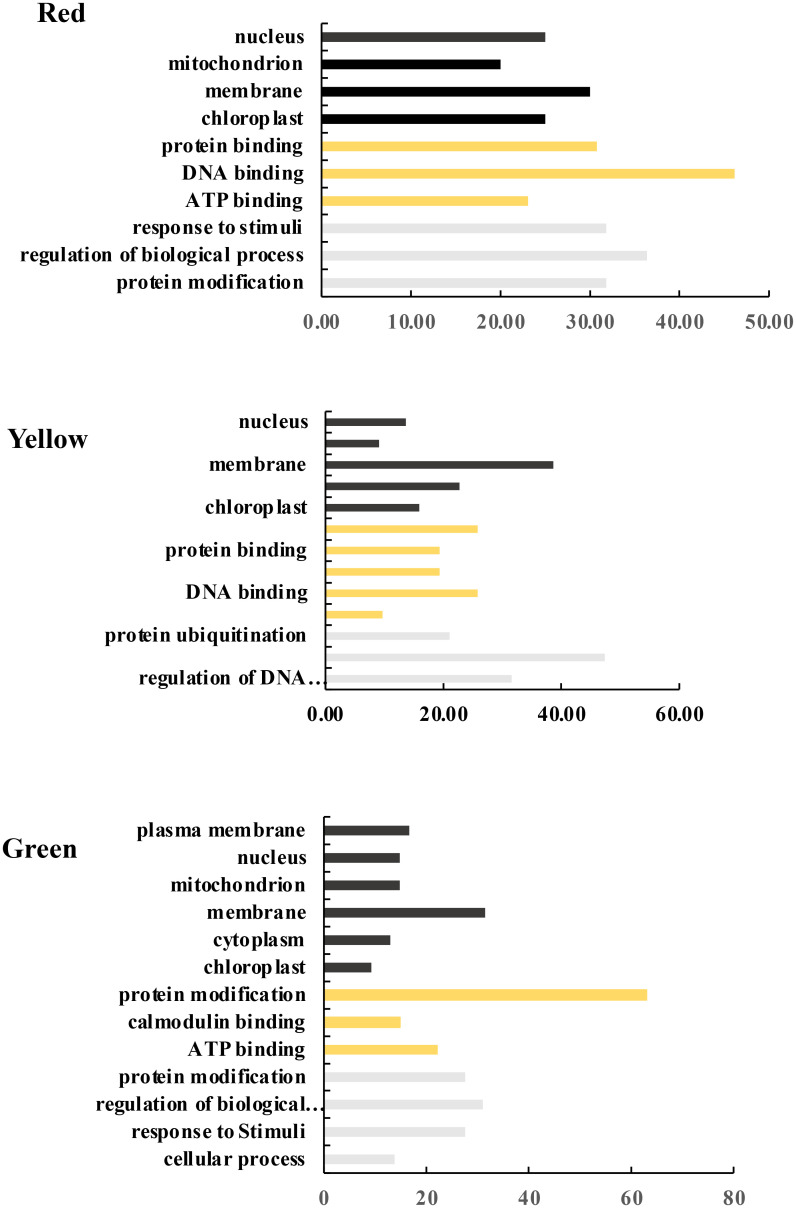
Enrichment of the modules associated with PVY resistance. The Gene Ontology enrichment was revealed in three modules of red, yellow, and green. GO-BPs, CCs, and MFs are indicated by black, orange, and gray boxes, respectively.

To discover the potential defensive mechanism in DEGs, GO analysis was used for the three modules. The GO terms were divided into three groups: biological process (BP), cellular component (CC), and molecular function (MF). The Fig 9 revealed the results of the GO analysis associated with the modules relevant to PVY-resistance. The top significantly enriched BP for the up-regulated transcripts included response to stimuli, regulation of biological process, and protein modifications. The most significantly CC for the up-regulated transcripts were nucleus, chloroplast, membrane, and mitochondria. The significantly enriched MF for up-regulated transcripts were protein binding, DNA binding, and ATP binding.

### Morphological and physiological difference among 10 cultivars under PVY infection

**Fig 10:**
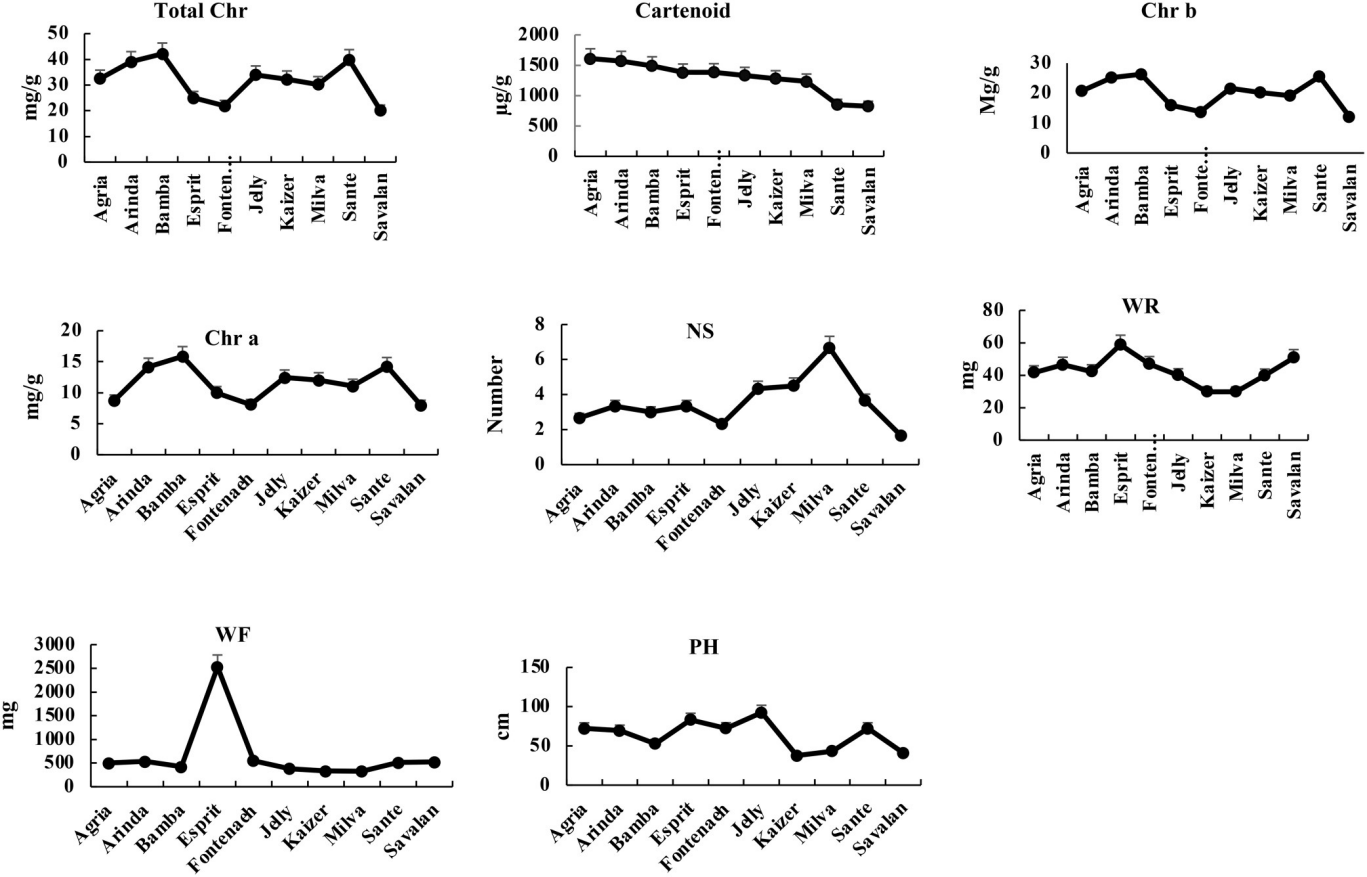
Morphological and physiological traits in 10 cultivars at 3 days after PVY infection.

**Fig 11:**
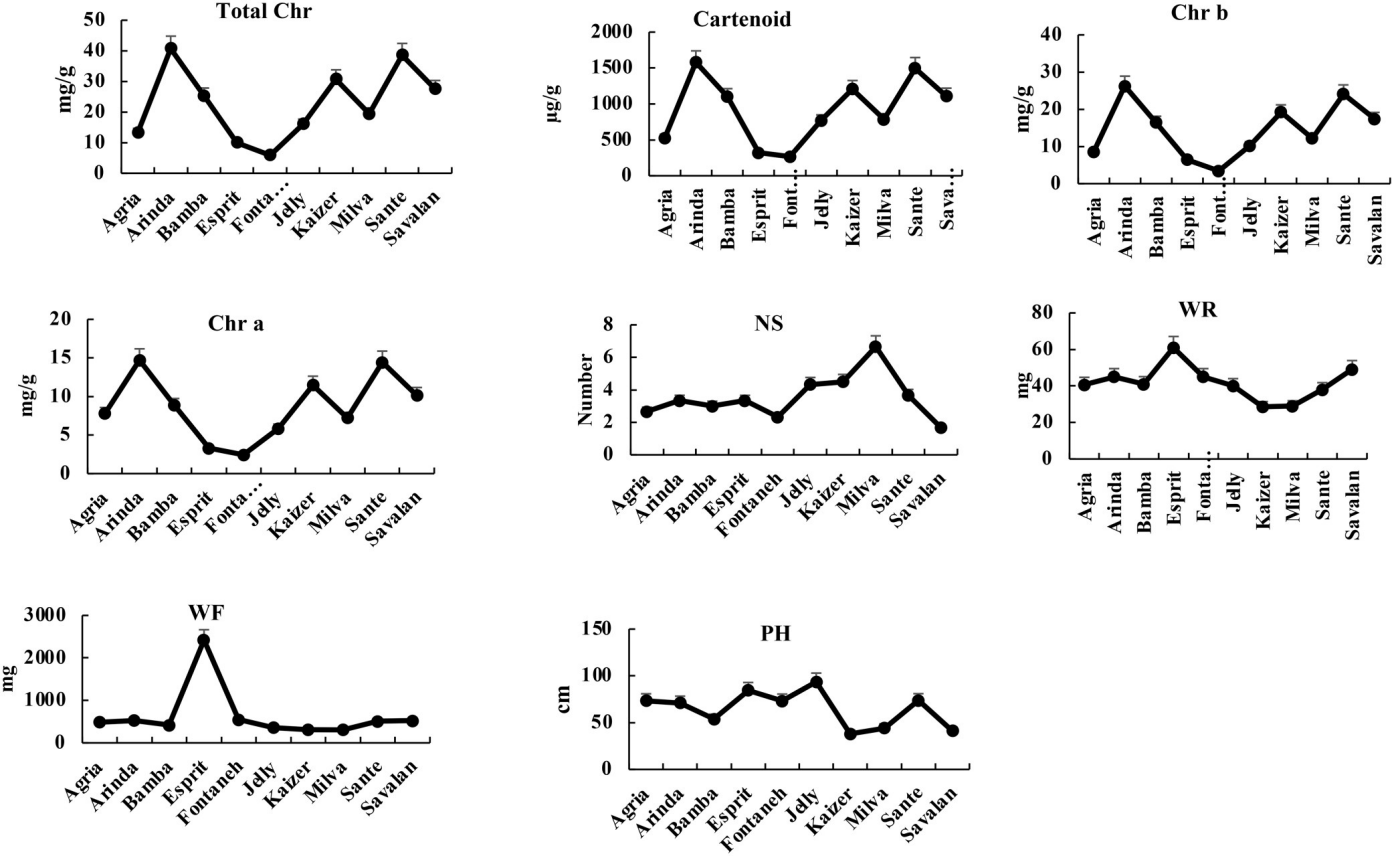
Morphological and physiological traits in 10 cultivars at 6 days after PVY infection.

After 3 days of PVY infection, the total chlorophyll ranged from 20.18 mg/g (Savalan) to 32.54 mg/g (Agria). The chlorophyll a ranged from 7.99 mg/g (Savalan) to 15.86 mg/g (Bamba) with an average of 11.46 mg/g among the 10 cultivars. The chlorophyll b varied from Fontaneh (13.76 mg/g) to Bamba (26.29 mg/g). The carotenoid content ranged from 824.88 mg/g to 1610.24 mg/g for Savalan and Agria, respectively. The maximum and a minimum number of stem with 6 and 1 belonged to Milva and Savalan, respectively. The average stem dry weight was 42.63 mg ranging from 29.81 mg (Milva) to 58.8 mg (Esprit). The fresh weight varied from Kaizer (327.83 mg) to Esprit (2529 mg). The plant height was 63.676 cm ranging from 37.66 cm (Kaizer) to 92.5 cm (Jelly) (Figs 10 and 11).

**Fig 12:**
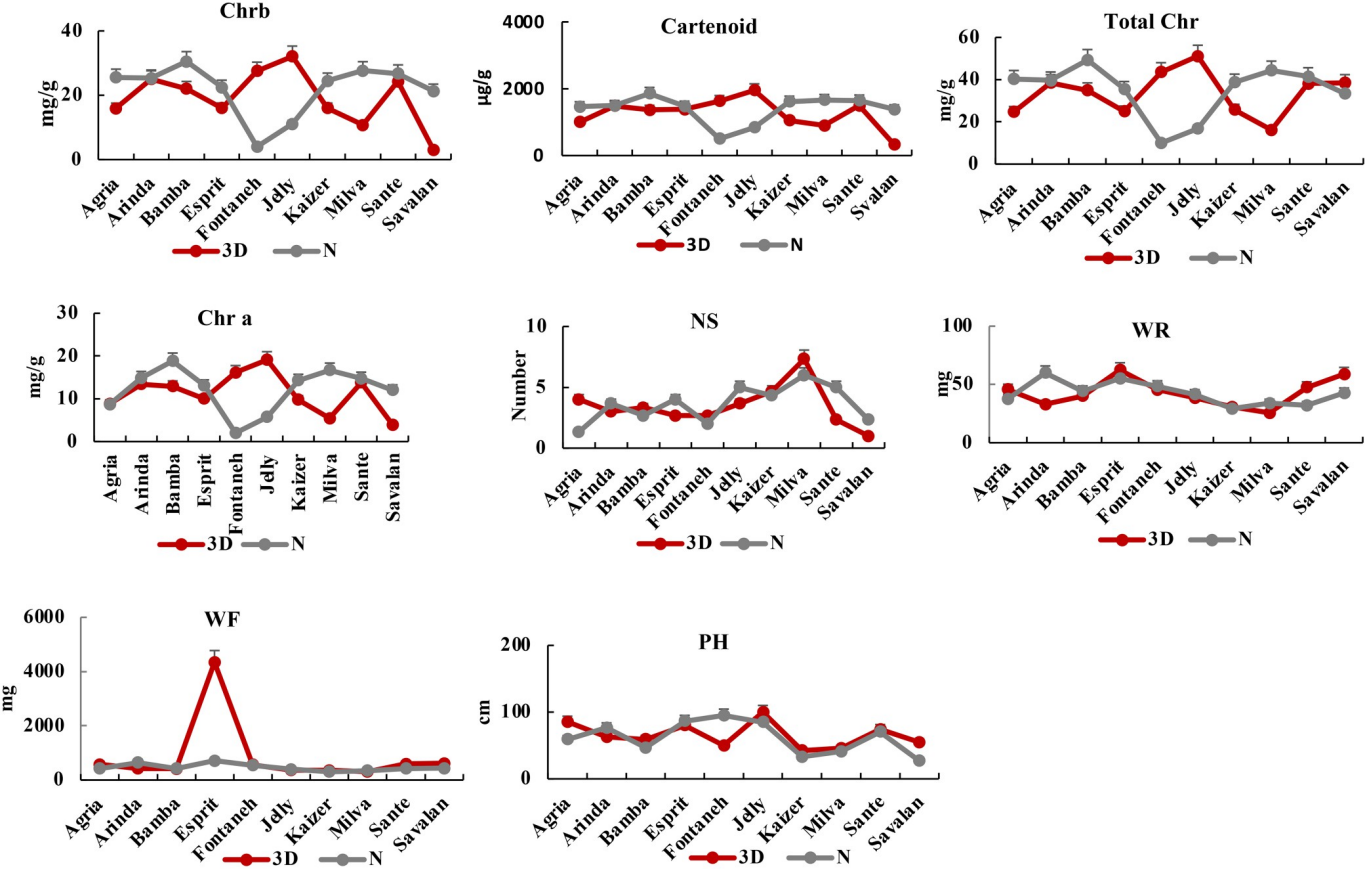
The interaction of morphological and physiological traits with genotypes among the 10 cultivars at 3 days after PVY infection.

**Fig 13:**
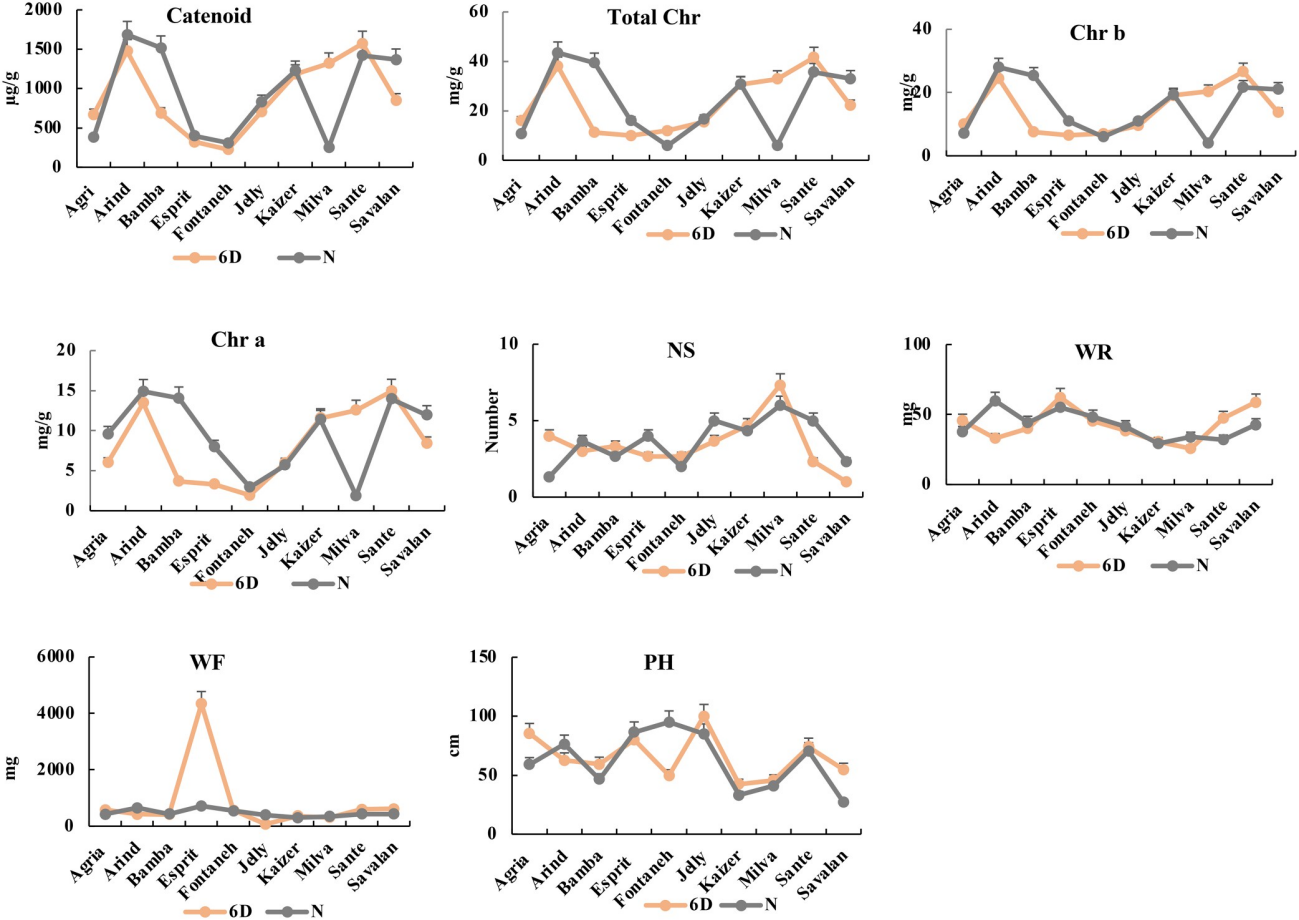
The interaction of morphological and physiological traits with genotypes among the 10 cultivars at 6 days after PVY infection.

After six days of PVY infection, the highest and lowest total chlorophyll belonged to Arinda (40.73 mg/g) and Fontaneh (5.87 mg/g). The chlorophyll a ranged from Esprit (3.3 mg/g) to Arinda (14.71 mg/g). The chlorophyll b varied from Arinda (26.27 mg/g) to Fontaneh (3.43 mg/g). The carotenoid content ranged from 320 mg/g to 1580.53 mg/g for Esprit and Arinda, respectively. The maximum and minimum number of stem with 6 and 1 belonged to Milva and Savalan, respectively. The average stem dry weight was 41.42m g ranging from 28.6 mg (Kaizer) to 61 mg (Esprit). The fresh weight varied from Kaizer (310 mg) to Esprit (2421 mg). The plant height was 64.678 cm ranged from 38 cm (Kaizer) to 93.6 cm (Jelly). The changes in morphological and physiological index indicated that the Fontaneh cultivar was the most sensitive cultivar among the 10 potato cultivars. However, Arinda, Jelly, Esprit, and Sante showed maximum chlorophyll and carotenoid contents as well as fresh weight, dry weight, plant height, and number of stem among the 10 cultivars (Figs 12 and 13). Cultivar selection for PVY resistance based on a combination of morphological and physiological traits and transcriptomic analysis might be possible and more effective in diverse environments. Further, high correlation between morphological and physiological traits can be indicative that the genetic variability have been maintained throughout the breeding process. However, the genetic distance based on the SNP markers, on an average, was higher than morphological, physiological, and transcriptomic analysis, which may reflect the effects of the environment on the performance of the cultivars.

## Discussion

Based on our results, GBS can accurately distinguish among commercial potato cultivars. In the current study, population structure and genetic diversity of commercial potato cultivars were assessed. The evaluation and assessment of genetic diversity in plants are one of the vital aspects in genetic improvement of potato. One of the most important requirements of potato breeding program is the understanding of the genetic diversity in germplasm collections and the relationships between genotype-phenotype for the basic understanding of adaptive traits. The SNP genotype calling of GBS approaches is typically more intense and precise for allelic dosage determination in plants, such as the tetraploid potato [33]. Using the GBS approach, marker discovery and genotyping of many samples can be multiplexed to decrease cost per sample. The SSR and SNP markers are becoming valuable resource to increase our insights on the genetic structure of potato populations, a necessary step for the conservation and future utilization of potato gene pools and for the recovery of alleles left behind by selective breeding. Such reservoir of alleles provides a powerful tool for breeders to undertake efficient breeding programs for the development of novel varieties best suited to new cropping systems and biotic and abiotic stresses. To the best of our knowledge, no studies have been performed and no data have been reported yet on commercial potato germplasm based on genotyping by sequencing. We performed a genome-wide diversity survey on a panel of 10 cultivars, representative of commercial germplasm, through genotyping-by-sequencing. By using a reference-based SNP calling pipeline, we developed an extensive catalogue of SNPs utilized to model population structure via clustering analysis and assessment relationships among commercial potato cultivars. In addition, the GBS approach was extensively utilized to develop SSR markers which is a more cost-efficient and faster method than Sanger sequencing without the need for SSR enrichment [34]. Further, the SNP genotype calling of GBS approaches is typically more intense and precise for allelic dosage determination in plants, such as the tetraploid potato [33]. Furthermore, our GBS results agreed with that of the WGCNA analysis which is an effective technique for categorizing the transcriptome data into co-expression modules to reduce the number of potential candidate genes.

### SSR mining using GBS

In this study, we applied GBS approach to generate SSR markers from 10 cultivars. Among the 3962 SSRs, the most frequent SSRs were dinucleotide repeats, representing 73.2% of all SSRs. Tri-nucleotides ranked second and represented 15.66% of all SSRs. Our results are in agreement with Yang et al. [35] who reported the highest number of di-nucleotides in spinach. In this study, AT-rich repeats were the most common repeat type especially for di-nucleotides. Also, our findings are in agreement with Cardle et al. [36] who showed that TA repeats are the most common repeat type for di-nucleotides. Previous survey has suggested that SSR with long core motifs are less frequent and shorter than mono-di-nucleotides. The SSR-enriched genomic libraries yielded only a few numbers of clones containing long types of repeats [37]. Furthermore, the most libraries were constructed containing di-nucleotide markers due to their higher frequency in the genomes and ease to isolate [38]. In our study, the newly developed SSR markers were located on different chromosomes. The chromosomes 2 and 11 had the most number of SSRs. Moreover, most common SSRs for di-nucleotides included TA and AT repeats. Previous survey has showed different distribution of SSRs among the 12 potato chromosomes [39]. Based on their research, the chromosome 1 displayed the highest densities of SSRs in the DM reference genome of potato. In the current study, the SSR mining was performed using GBS method and has proven to be effective in potato and other plants with a large genome size or those without a complete genome reference. Further, we developed two novel sets of molecular markers namely SSR and SNP markers. Previous survey showed that developing SSRs by NGS data can be economically cost effective [40]. In addition, unlike SSRs, the SNPs are bi-allelic with a maximum PIC value of 0.5. Due to their low PIC values, the utilization of SNP markers has been reduced. For example, a study on wheat showed that out of 76 SNP markers only 22 were polymorphic [41].

### Population structure and genetic diversity

The DAPC analysis identified a clear genetic distinction across the surveyed potato germplasm, dividing the germplasm into distinct clusters related to their genetic structure related to ploidy and taxonomy of the cultivars. Based on their different taxonomic backgrounds, the 10 commercial potato cultivars were divided into three clusters. In terms of genetic diversity, according to the analysis of PCA, DAPC and neighbor-joining tree for the SNP markers, the cultivars were approximately divided into the same numbers of clusters with similar broad patterns. However, the number of cultivars categorized into the clusters varied. Based on our results, dendrograms were made with different methods in DAPC and Nei which these outcomes were not strange. Our results of DAPC, GD, and phylogenetic tree agreed with Deperi et al. [42] in potato. Previous survey showed that resistant cultivars constitute a valuable source of new alleles for various complex traits utilized in breeding programs, particularly when wide genetic base is required [43]. Further, it has been reported that the GBS on a large collection of autotetraploid potato cultivars.

It is to be noted that the DAPC and PCA analysis grouped the cultivars with low levels of admixture. This finding was evident from the NJ and MSN. The NJ clustering and MSN revealed that clustering of the cultivars tend to be based on their geographical origin [44]. We utilized DAPC, PCA, NJ cluster analysis, and MSN analysis to truly understand the genetic structure of these cultivars. Based on another survey, DAPC was slightly better than STRUCTURE and it achieved appropriate separation among groups in potato [45]. The outcome of DAPC, PCA, and MSN included low admixture within the clusters, indicating genetic relatedness among individuals from low admixture between the clusters. These methods (fineRADstructure and DAPC) have better met our expectations, as it was observed that the cultivars are not well clustered via NJ method as compared to other mentioned methods (GD, PCA, and MSN). Based on the DAPC and FineRADstructure results, individuals of cultivars in three clusters were separately grouped. Based on our findings, the 10 cultivars were grouped in three clusters using genetic markers.

The clustering pattern of cultivars with similar geographical origin were grouped exclusively in a single or two clusters [46]. The identification of genetic diversity among individuals of cultivars could be used to successfully initiate a new crossing program. To classify our available germplasm into various/ heterotic groups, it is important to know about the current genetic diversity, which is crucial for hybrid/cross-breeding programs for potato. Previous survey showed that the close relationship between molecular genetic variability with some cultivars studied in this research could be due to the close geographic sources of these cultivars [46].

### FineRADstructure analysis

Our results identified at least three genetic clusters in the germplasm pool used in this study. Our results revealed that had lower co-ancestry for the group I as compared to group III. The hybrid presence of group I and III revealed evident signs of population admixture in two groups. Based on another result of the fineRADstructure, selected individuals by deriving a co-ancestry matrix, pairs of individuals shared the most similar haplotypes [47]. Co-ancestry measures genetic similarity of two individuals; higher co-ancestry indicates less divergence of two individuals [48]. The structure provided by the fineRADstructure analysis approximately corroborated with the results of the DAPC analysis, placing the same individuals in the same three clusters. These methods (fineRADstructure and DAPC) have better met our expectations, as it was observed that the cultivars are not well clustered via NJ method as compared to other mentioned methods (GD, PCA, and MSN). Based on the DAPC and fineRADstructure results, individuals of cultivars in three clusters were separately grouped. Due to the limited utilization of certain germplasms from various regions of the world, the genetic base of many cultivated potato varieties has become limited. As a result, knowledge of genetic diversity and kinship of common potato cultivars are essential to identify differentiated lines and produce seed tubers with high genetic purity used for selection of desired lines in the crossing processes. The narrow genetic base of cultivated potato can be expanded through the crossing of heterosis parents. The presence of admixture is an opportunity to detect selection for crossing. As a result, hybrid presence could be used for higher heterosis in potato genetic improvement programs. Admixture can occur when new favorable alleles are introduced into elite germplasms [49]. Based on our results, the Milva and Esprit have putative potential for use in potato breeding programs.

### Morphological and physiological indices among cultivars under PVY infection

The changes in morphological and physiological index indicated that the Fontaneh cultivar was the most sensitive cultivar among the 10 potato cultivars. However, Arinda, Jelly, Esprit, and Sante showed maximum chlorophyll and carotenoid contents as well as fresh weight, dry weight, plant height, and number of stem among the 10 cultivars. Our findings showed that some of the cultivars had low chlorophyll content in the infected plants as compared to the control plants. Another survey indicated that the contents of chlorophyll a and b of infected leaves were more than uninfected leaves in ‘Ergenekon’ and ‘497’ in pepper [48]. In this study, most cultivars had lower fresh weight, dry weight, plant height, and number of stems of infected plant lower than the control. However, Esprit gave the highest fresh weight and dry weight as compared the other cultivars. Other findings on sorghum [50], chickweed [51], E. makinoi [52], cucurbit [53], sugar beet [54], and S. nigrum [55] support our findings. In pepper, Tobacco mosaic virus (TMV) infection caused reduction in shoot fresh and dry weight [48].

### WGCNA analysis for PVY resistance

In this survey, three selected genes with a high degree of connectivity, based on the MCC in the yellow, red, and green modules, were correlated with PVY resistance. In the face of pathogens, plants used defense strategies, including: firstly, alkalization in apoplast is as one of first response to pathogen. Secondly, chloroplast protein degradation and generation of protein-derived peptides may involve in forming defense proteins [56]. According to the WGCNA analysis, the *StTMEM161A* gene possesses a high correlation in the module. The overexpression of StTMEM161A leads to reduced levels of oxidant-induced DNA damage and apoptosis. Previous study indicated that TMEM161A plays a role in protecting against oxidative stress [57]. This gene is activated by calcium as a chloride channel. Chloride channels are a superfamily of unknown chloride-specific ion channels. These channels may conduct various ions, but these channels are named as chloride because its concentration in the cell is much higher than that of other anions. The anionic chloride affectes the pH of the apoplast. Overall, the pH is as an important modulator of plant gene expression which interacts with pathogen attack in integrating cellular monitoring of the extracellular compartments. Apoplast alkalization is one of the first plant response to effectors along with rise in cytosolic Ca^+2^ because various pathogens rely on apoplast acidification to colonize their host [58]. The apoplastic pH affects the development of disease and the physical and chemical properties of the cell wall. Moroz et al. [59] reported that an alkalization response against *Phytophthora infestans*, *Spongospora subterranean*, *Verticillium dahlia*, and *Colletotrichum coccodes* can be used as an effective marker to survey early stages of defense response in potato. Previous study has shown that transient apoplast alkalization associated with the degree of resistance to fungal pathogens during pathogen-host plant interactions was observed on barely under powdery mildew fungus [60].

Chloroplasts contain a large amount of protein, and rapid degradation during stress is a key process that can provide nutrients for translocation to growing organs. Previous study showed that pathogen attack can damage plant tissues. Chloroplasts can respond to stress and changes in the environment by producing reactive oxygen species (ROS) [61]. Upon pathogen attack, ROS signaling induces protein, providing additional sources of antimicrobial proteins during biotic stress response [62]. This argument revealed that chlorophyll catabolism is yet the ambiguous process in plants. It has been shown that DUF538 induction is one of the first defensive responses of plants to stresses [63,64]. The DUF538 is up-regulated across the hypersensitive response (HR), and the production of reactive oxygen species (ROS) in RKN-resistant genotype, *M*. *arenaria*, to *A*. *stenosperma* is induced through the involvement of DUF538 in antioxidant activities [65]. Previous survey indicated that a protein of unknown function containing a DUF538 domain had high expression in the PVY infected potato plants [66]. Some reports revealed that DUF538 is induced under different pathogen invasion. DUF538 transcripts or proteins were increased in response to fungal infection through activating plant antioxidative system and other common components in response to plants exposed to different stresses [67–69]. Earlier survey demonstrated that DUF538 is expressed in the nematode resistance peanut [70].

In this study, comparison of the bioinformatics data with the experimental findings suggested that the StDUF538 proteins could degraded chlorophyll molecules. The relationship of StDUF538, gene expression level with the chlorophyll content of the PVY-infected leaves is probably resulted in response to biotic stress. Further, *StTMEM161A* and *StGTF3C5* genes are fundamental mediator in pH apoplast and regulation of tRNA biogenesis in growth and response to pathogens in resistant cultivar, respcetively. Based on our results, Esprit cultivar had the maximum expression of *StDUF538*, *StTMEM161A*, and *StGTF3C5* genes. Further, Esprit possess the low chlorophyll content at the 3 and 6 days after PVY infection as well as Esprit had the maximum fresh weight and dry weight under 3 and 6 days after infection. Thus, Esprit can be suggested as a resistant cultivar against PVY infection.

## Conclusion

Our results indicated that the GBS analysis on commercial cultivars is an effective strategy for identifying population structure and genetic diversity. Further, the GBS data provided a more comprehensive characterization of the groups and helped to comprehend the population’s structure.

Based on the GBS and WGCNA, six modules highly correlated with individual and PVY resistance genes. Within modules, three hub genes were identified and analysis of gene expression of StDUF538 and low chlorophyll content indicated the pivotal role of chloroplast in PVY resistance response, confirming the WGCNA results. Overall, the combination of transcriptome, morphological, physiological, and genomic data might highlight the gene networks and molecular mechanisms involved in PVY resistance. Based on methodologies used in the current work, similar population structures were identified among the 10 cultivars, corresponding to genetic backgrounds in the same clusters as well as facilitating cultivar selection in potato breeding programs. In this study, the Esprit cultivar had a high expression of *StDUF538*, *StTMEM161A*, and *StGTF3C5* genes, along with maximum fresh weight and dry weight at 3 and 6 days after PVY infection, making it a suitable candidate for development of PVY resistant potato cultivars. Results of this survey provide useful information for genetic improvement and plant conservation programs, although further surveys are required for a more accurate assessment of genetic diversity and phenotypic traits of potato.

## Supporting information

S1 Table Primer sequences used for real time RT-PCR in this study. (DOCX)
