# Supplementary material for: Genotyping-by-sequencing and weighted gene co-expression network analysis of genes responsive against Potato virus Y in commercial potato cultivars
Source: PLoS One. 2024 May 24;19(5):e0303783. doi: 10.1371/journal.pone.0303783 (PMC11125566; doi:10.1371/journal.pone.0303783)
Supplement: S1 Table — (DOCX) [file pone.0303783.s001.docx]

**Table S1. Primer Sequences Used for Real Time RT-PCR in this Study.**

| No. | Primer name | Sequences |
| --- | --- | --- |
| 1 | F: StTMEM161A  R: StTMEM161A | TGCCTGCATCAATGGTAC  ACAATAGATCAAGATACACAC |
| 2 | F: StDUF538  R: StDUF538 | ATTCATCTGTATTACGCTTC  GTTGATCACACTAGAATTTATC |
| 3 | F: StGTF3C5  R: StGTF3C5 | TGTTGAAGAGGAGATGGATGC  CATGATGTCTTAGTAGTCTTCA |
| 4 | F:StEFα1  R:StEFα1 | AGATGGTCAGACCCGTGAAC  CCTTGGAGTACTTCGGGGTG |
